# Advances in Serum-Free Suspension Culture Technology for Animal Cells and Their Applications

**DOI:** 10.3390/vaccines13111109

**Published:** 2025-10-29

**Authors:** Wenna Ji, Ziyi Chen, Jinyu Zhou, Xinyu Yue, Zilin Qiao, Jiamin Wang

**Affiliations:** 1 Ministry of Education Engineering Research Center for Key Technologies and Industrialization of Cell-Based Vaccines, Northwest Minzu University, Lanzhou 730030, China; wennaji0721@163.com (W.J.); 19911595109@163.com (Z.C.); qiaozilin@xbmu.edu.cn (Z.Q.); 2 Gansu Animal Cell Technology Innovation Center, Northwest Minzu University, Lanzhou 730030, China; jyzhou919@163.com; 3 State Ethnic Affairs Commission Key Laboratory of Bioengineering and Technology, Northwest Minzu University, Lanzhou 730030, China; 15547202798@163.com; 4 Gansu Provincial Bioengineering Materials Engineering Research Center, Lanzhou 730030, China

**Keywords:** suspension culture, serum-free medium, novel cell lines, genetic engineering, biological products

## Abstract

Serum-free suspension culture technology for animal cells involves the division and proliferation of cells in serum-free medium as single cells or cell clusters within shaking flasks or bioreactors. This approach enables large-scale cell culture, enhances the yield and quality of biopharmaceuticals, reduces costs, and broadens the applications of animal cells. Serum-free suspension culture of adherent cells (e.g., Madin–Darby canine kidney (MDCK), Chinese hamster ovary (CHO), Vero, baby hamster kidney (BHK-21), and human embryonic kidney (HEK293) cells) has been successfully achieved through direct or indirect adaptation, medium optimization, and genetic engineering. Additionally, novel suspension cell lines, such as duck embryonic stem (EB66) and human retinoblastoma (PER.C6) cells, have been developed as potential new substrates for biopharmaceutical production. This review examines animal cell suspension culture technology and its applications in viral vaccines, recombinant proteins, and monoclonal antibodies, providing insights into the development and utilization of this important technology.

## 1. Introduction

Animal cell culture involves the isolation of cells from animal tissues or organs and their cultivation in vitro under conditions that replicate the physiological environment of the body (including sterility, temperature, pH, osmotic pressure, nutrition, and gas composition) to support survival, proliferation, and the maintenance of specific structures and biological functions [1]. Harrison’s pioneering work marked the beginning of cell culture technology, a discipline that has evolved for more than a century [2]. Since the late 1940s, the application of animal cell culture for virus production has been recognized as the cornerstone of modern vaccine technology. Initially developed for vaccine manufacturing, cell culture technology has become a key aspect of biopharmaceutical production. The number of biopharmaceuticals produced using animal cell culture technology, along with their demand, has rapidly increased [3]. Animal cell culture is now widely utilized in biomedical research, drug development, vaccine production, and gene therapy, forming an indispensable component of modern biotechnology.

**Figure 1:**
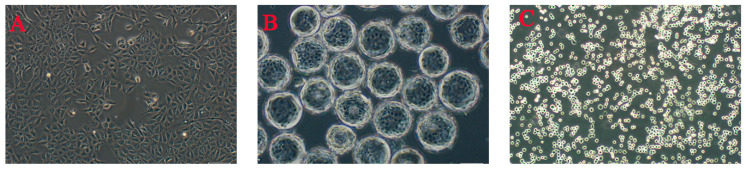
MDCK cells cultured by various methods in our lab ((**A**) monolayer adherent culture, P68; (**B**) microcarrier culture, Cytodex 1, P68; (**C**) serum-free whole suspension culture, P116).

According to the culture method, animal cell culture can be divided into two major categories: adherent and suspension. The former is primarily suitable for anchorage-dependent cells, which exhibit interdependent relationships and require attachment to a biocompatible solid surface for spreading and growth (Figure 1A). This process typically involves the use of flasks, cell factories, microcarriers, or fixed-bed bioreactors. Adherent culture in flasks is simple to operate and facilitates observation, making it widely used in laboratory research. However, adherent cells display contact inhibition when they reach confluence, and cell growth is suppressed after formation of a dense monolayer [4], directly limiting the usable surface area. To address this limitation, a mature and practical large-scale culture method—microcarrier culture—was developed (Figure 1B). In the mid-20th century, Van Wezel [5] pioneered microcarrier cell culture, introducing a new technology for high-yield cultivation of adherent cells. Commonly used microcarriers include collagen-coated, porous, gelatin-based or collagen-based, and biodegradable types; these microcarriers are characterized by a large specific surface area, good biocompatibility, and compact size [6]. However, no single carrier is suitable for all cell types. Additionally, some studies have shown that carriers may adsorb certain nutrients from the culture medium, substantially affecting cell growth. For example, the potential cytotoxicity of certain microcarriers used as scaffold materials for implantable cells requires investigation [7]. Since its development in 1960, suspension technology for animal cells has become the preferred model for large-scale cell culture [8]. This approach allows cells to proliferate in suspension within the culture medium as single cells or as small aggregates (Figure 1C), overcoming the limitations of adherent culture and offering multiple advantages: (1) uniform nutrient distribution through single-cell suspension and (2) significantly improved growth rates and culture efficiency, enabling industrial-scale production. Compared with microcarrier-based systems, serum-free suspension culture is more suitable for large-scale production of biopharmaceuticals such as vaccines and monoclonal antibodies [9].

Based on whether serum is added to the medium, cell culture can be classified as serum-containing or serum-free. In serum-containing culture—whether adherent or suspension—serum provides inherent advantages that support cell growth. It supplies essential nutrients for cellular metabolism and contains numerous active growth factors, such as epidermal growth factor, insulin-like growth factor-1, and transforming growth factor-β, which regulate cell proliferation and survival. These factors can adsorb toxic substances in the medium and reduce shear stress–induced damage, thereby improving cell viability and proliferation efficiency [10]. However, serum is a naturally derived mixture containing undefined components—such as unknown proteins and cytokines—that may vary among batches. This variability can directly affect the reproducibility of experimental results. More importantly, serum from animal sources (e.g., fetal bovine or calf) might introduce exogenous contaminants, posing potential safety risks in clinical-grade cell therapy and vaccine production. The development of serum-free media addresses these limitations by replacing serum with a precisely defined combination of chemical additives. Each component is known in terms of type and concentration, eliminating ambiguity and batch-to-batch variability while allowing standardization of the culture process. The absence of animal-derived materials also completely removes the risk of exogenous contamination, meeting safety requirements for clinical applications [11].

This paper systematically outlines the complete technological chain of total suspension culture technology, spanning from cell adaptation, serum-free medium optimization, and genetic engineering modifications to large-scale bioreactor cultivation and monitoring. It comprehensively covers the development and application of multiple novel suspension cell lines, including Duck Embryonic Stem (EB66), Human Retinoblastomal (PER.C6), and Muscovy Duck Embryonic (AGE1.CR), thereby overcoming the limitations of previous approaches that primarily focused on traditional cell types. Simultaneously, it emphasizes integrating Process Analytical Technology (PAT) and Quality by Design (QbD) principles into culture process optimization. The work extensively addresses production applications across multiple biopharmaceutical categories, including viral vaccines, recombinant proteins, and monoclonal antibodies. Ultimately, it bridges technological breakthroughs with industrial implementation challenges, embodying holistic perspective spanning laboratory research to large-scale production. This approach demonstrates significant systematic and engineering guidance value.

## 2. Suspension Culture Technology

A key aspect of suspension culture technology is the adaptation of adherent cells to suspension growth, which requires cells that normally grow adherently to gradually acclimate to proliferation in a suspension format. The core of this process lies in progressively modifying the growth environment to promote suspension adaptation. Current approaches to adaptation include direct or indirect methods, medium optimization, and genetic engineering. Several fully suspended cell lines with stable genetic characteristics and high production efficiency have been successfully established, including Madin–Darby canine kidney (MDCK) cells, Chinese hamster ovary (CHO) cells, Vero cells, baby hamster kidney (BHK-21) cells, and human embryonic kidney (HEK) 293 cells. Adaptation to suspension culture is a critical step in establishing fully suspended cell systems. By applying suitable adaptation methods and optimizing culture conditions, cell adaptability and growth performance can be enhanced, providing a stable platform for subsequent biopharmaceutical production.

### 2.1. Direct and Indirect Methods

**Figure 2:**
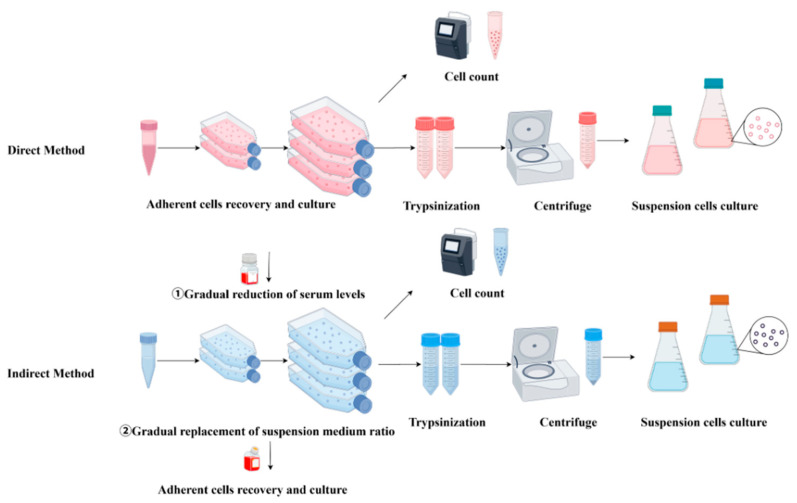
Direct and indirect methods of suspension adaptation.

Before cells can be adapted to single-cell suspension culture, they are first acclimated to serum-free suspension medium using either a direct or an indirect method. The direct method involves immediately replacing the original adherent culture medium with suspension culture medium. In contrast, the indirect method involves gradual transition to suspension culture by mixing serum-containing adherent culture medium with serum-free suspension culture medium in varying proportions (Figure 2), a process that can require up to 2 months for full adaptation [12]. For example, Paillet et al. [13] used the gradual method, which required 120 days to adapt VeroE6 cells to suspension growth in a custom-made serum-free medium; they ultimately obtained S-Vero cells capable of single-cell growth. Wielink et al. [14] utilized a protocol in which serum-containing medium was progressively replaced with serum-free medium after 7–12 passages. MDCK cells grown in shaking flasks at a density of 2.2 × 10^6^ cells/mL successfully adapted to suspension growth in commercial serum-free medium. Similarly, Lee et al. [15] used a stepwise adaptation method to produce Vero cells capable of suspension growth in serum-free OptPRO medium, achieving an adenovirus yield 1.5 times higher than that of adherent Vero cells. However, not all animal cells can be directly adapted to suspension growth. In such cases, methods such as medium optimization and genetic engineering are necessary to facilitate suspension adaptation, thereby enhancing cellular adaptability and growth efficiency.

### 2.2. Serum-Free Medium Optimization

Serum-free suspension culture medium is specifically formulated for cells grown in suspension, free of serum components; it is designed to provide a defined and stable environment that supports cell growth and metabolism. The key characteristics of this medium include well-defined composition, high reproducibility, and reduced risk of exogenous contamination. The development of serum-free media suitable for suspension cell growth and viral replication can increase cell density, improve viral titer, and facilitate downstream purification of bioproducts. The composition of such medium typically includes a basal medium (e.g., Dulbecco’s modified Eagle medium/F12), amino acids, vitamins, inorganic salts, trace elements, buffers, antioxidants, growth factors, and hormones. These components act synergistically to supply essential nutrients and growth signals, promoting cell proliferation and differentiation [16]. Hideo Miki [17] reported that CHO cells cultured in serum-free medium supplemented with insulin-like growth factor-1 analogs and lysophosphatidic acid exhibited increased specific growth rates. Insulin-like growth factor-1 has been shown to stimulate cell proliferation through the extracellular signal-regulated kinase/mitogen-activated protein kinase signaling pathway and to promote cell survival via the phosphoinositide-3-kinase/protein kinase B pathway. Rourou et al. [18] cultured fully suspended Vero S cells in IPT-AFM medium with reduced Ca^2+^ and Mg^2+^ contents, achieving cell densities comparable to those of adherent Vero cells grown on Cytodex 1 microcarriers; the Vero S cells produced higher rabies virus titers. Serum-free or chemically defined media can also be customized to meet the specific needs of various cell types (Table 1). Examples include animal-free media, protein-free media, and xeno-free media. Litwin et al. [19] successfully established suspension cultures of Vero cells in an internally developed serum-free medium containing insulin, bovine serum albumin, and commercial serum substitute concentrates, reaching densities of 10^6^ cells/mL within 20–30 days. Li et al. [20] investigated the role of heparin in the serum-free suspension adaptation of CHO-TS 28 cells, demonstrating its ability to promote cell growth and product secretion, maintain the original specific growth rate (μ) and specific monoclonal antibody productivity (qMAb), and enhance antigen-binding activity. In monoclonal antibody production, recombinant protein manufacturing, vaccine development, and cell biology research, the optimization of serum-free suspension media for animal cells is essential to improve production efficiency and product quality. With respect to product stability: (1) Serum-free media eliminate the undefined natural mixtures found in serum, thereby removing variations in product quality due to fluctuations in unknown proteins and cytokines across serum batches. (2) The absence of animal-derived components completely avoids potential exogenous contaminants, such as viruses and mycoplasmas, that may be present in serum, preventing structural damage to the final product. (3) The low-protein composition simplifies downstream purification, reducing product loss and minimizing structural alterations during purification, which ensures stability from upstream to downstream processing. Through medium optimization, these properties collectively enhance product stability and consistency while significantly increasing cell growth density, product expression levels, and process reliability. Although initial costs for media, additives, and equipment may be higher, such optimization provides substantial long-term benefits by greatly improving cell yield, product quality, and production efficiency, while strengthening overall product stability and consistency—ultimately resulting in considerable economic advantages.

The optimization and formulation of serum-free suspension culture media are primarily achieved through two approaches. The first is an experimental design-based optimization method. Design of experiments enables the simultaneous optimization of multiple components and their interactions, thereby simplifying experimental procedures, improving efficiency, and reducing both the number of experiments and overall workload. Progressing from full factorial designs to fractional factorial designs, and further to more advanced models such as the Plackett–Burman method and response surface methodology, researchers can identify key factors and partial interactions, thus minimizing experimental requirements. For example, Cui et al. [21] utilized a novel experimental design—the superlative box design—to optimize the adaptation of CHO cells from adherent culture to serum-free suspension culture. This process involved gradually reducing serum levels to acclimate the cell line to serum-free medium, combined with agitation at varying speeds in shaking flasks to transition cells from adherent to suspension growth. Response surface analysis was then used to optimize process conditions. A quadratic curve model calculated the optimal cell seeding density as 7.02 × 10^5^ cells/mL. The second approach is based on cellular metabolism. This method involves continuous monitoring of nutrient levels—such as glucose, amino acids, vitamins, fatty acids, and trace elements—throughout cell culture. By continuously replenishing rapidly depleted nutrients, more stable conditions can be maintained during continuous culture.

### 2.3. Genetic Engineering

A key trend in the development of suspension cell culture technology is the use of genetic engineering to create more efficient suspension cell lines with enhanced viral yield and improved vaccine quality. Techniques such as CRISPR/Cas9 gene editing, overexpression vector construction, targeted gene knockout, and gene transfection via liposomes or viral vectors can be utilized to modify cellular growth characteristics and metabolic pathways, making them better suited for suspension culture. For example, specific guide RNAs (gRNAs) can be designed to direct Cas9 nucleases for precise gene knockout or insertion. Knocking out genes associated with adhesion can reduce anchorage dependence, whereas inserting genes linked to suspension growth can improve suspension capacity (Table 2).

For example, Backliwal et al. [30] reported that overexpression of the gene encoding the cyclin-dependent kinase inhibitor CDKN1B during the G0/G1 or G2/M phase—when cells are typically larger and more metabolically active—effectively slowed the growth rate and increased the specific production rate in HEK293 X suspension cells. Pech et al. [31] found that reduced E-cadherin expression and increased AMPK expression in MDCK cells under suspension culture conditions jointly influenced cell adhesion, metabolism, and autophagy, enabling adaptation to the suspension growth environment. Chu et al. [32] demonstrated that high expression of the *Siat7e* gene enhanced the growth rate of MDCK suspension cells by promoting autocrine activation of the receptor tyrosine kinase c-Met in the epithelial–mesenchymal transition pathway and reducing cell adhesion. Dai et al. [33] showed that reduced PABPC1 expression may inhibit apoptosis and facilitate suspension culture in BHK-21 and CHO-K1 cells. Overall, regulating the expression of cell adhesion-related genes may be a viable strategy to promote adaptation to suspension growth.

Genetically engineered suspension cell lines used for vaccine and recombinant protein production are indispensable, yet their application carries unique risks and stringent regulatory requirements. Key concerns include potential tumorigenicity from residual host cell DNA, immunogenicity due to non-human glycosylation or host proteins, viral contamination introduced during production, and genetic instability resulting from long-term culture (e.g., target gene loss, silencing, or mutation) [34]. Therefore, global regulatory frameworks mandate the establishment of a multi-layered defense system to ensure the safety, efficacy, and batch-to-batch consistency of final products. This system requires the creation of a fully characterized cell bank, validation of the production process’s ability to remove impurities, rigorous product characterization analysis, and the provision of genetic stability study data.

## 3. Large-Scale Cultivation in Bioreactors

Animal cell bioreactors are systems designed to replicate the internal environment of animals, enabling biological cultivation in vitro. They are advanced technological platforms that integrate multiple disciplines, including mechanical engineering, fluid dynamics, control systems, and biology. Key control parameters include temperature, dissolved oxygen (DO), pH, fluid dynamics, agitation speed, aeration sparger system, bioreactor diameter-to-height ratio, nutrient concentrations, and levels of metabolic byproducts. The primary objective is achievement of high-density cell growth for the efficient production of pharmaceutically valuable products such as enzymes, monoclonal antibodies, and vaccines. Research has shown that both bioreactor type and associated process parameters have substantial effects on cell metabolism and product yield. Therefore, careful optimization of bioreactor selection and culture parameters is needed to improve cell growth efficiency, enhance viral yields, and reduce production costs.

### 3.1. Reactor Types

**Figure 3:**
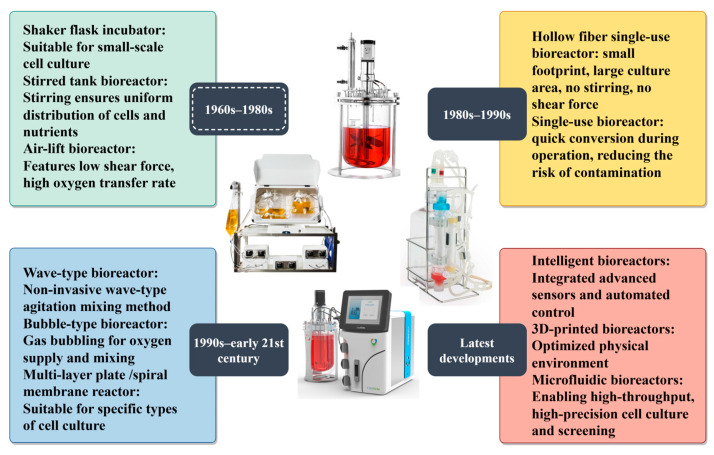
Bioreactor development history.

Based on the type of cells being cultured, bioreactors can be mainly categorized into adherent and suspension types. Adherent bioreactors include hollow-fiber and fixed-bed systems, whereas suspension bioreactors include stirred-tank and air-lift designs. The stirred-type bioreactor is one of the most widely used designs, applied in both industrial production and laboratory research [35]. However, the shear forces generated in stirred-type systems can cause cell damage, whereas non-stirred reactors produce lower shear forces, offering a substantial advantage for animal cell culture. For example, Lou et al. [36] developed a 5-L wave-type bioreactor for the large-scale production of recombinant human thyroid peroxidase. The resulting thyroid peroxidase exhibited good sensitivity and specificity when tested with human serum samples, demonstrating promising application potential. Tapia et al. [37] inoculated adherent or suspension MDCK cells into a single-use hollow-fiber bioreactor (PRIMER HF), achieving high-titer production of influenza H1N1 virus. The cell-specific virus yield was comparable to that obtained with conventional stirred-tank bioreactors. Conventional bioreactors have undergone substantial design improvements; new types continue to emerge, including single-use bioreactors, three-dimensionally printed bioreactors, microfluidic bioreactors, and membrane bioreactors (Figure 3). Conventional bioreactors allow precise environmental control and facilitate experiments using two-dimensional tissue models. However, their inability to replicate in vivo systems and accurately reflect real cellular characteristics and tissue morphology has led to the development of three-dimensional systems. Three-dimensional bioreactors enable improved spatial distribution and more complex tissue structures. For instance, Schneider et al. [38] developed a three-dimensionally printed bioreactor with a 90-mL working volume, integrating pH and DO sensors as well as optical bio-monitoring modules, which successfully supported high-density suspension culture of CHO cells. Microfluidic bioreactors are miniaturized culture devices that incorporate microfluidic technology. Their core function is the precise control of microfluidic flow (volumes ranging from nanoliters to microliters) through micrometer-scale channels, enabling fine regulation of the cellular microenvironment. The vertical motion flow reactor is a novel membrane bioreactor incorporating Shirasu porous glass membrane distributor technology, which generates highly uniform microbubbles. By efficiently supplying oxygen through the Shirasu porous glass membrane distributor and reducing DO aeration flow to minimize foam formation, the vertical motion flow reactor has been shown to support high-density culture of CHO cells.

### 3.2. Real-Time Monitoring

Real-time and near-real-time monitoring of cell culture processes are essential for the development of process analytical technologies in upstream bioprocessing. These monitoring systems provide rapid feedback on process disturbances, enabling timely interventions that can prevent batch failures. In the past, real-time monitoring in bioreactor processes was limited to parameters such as pH, DO, temperature, cell growth, and metabolic products. To reduce sampling errors and increase throughput, advanced real-time and near-real-time instruments have been developed. Examples include dielectric spectroscopy, integrated online high-performance liquid chromatography, and nanofluidic devices, which can assess cell growth, cell health, and product quality [39]. Berry et al. [40] used Raman spectroscopy and multivariate analysis to develop cross-scale predictive models for growth and metabolite levels in CHO cell cultures. Models for glucose, lactate, and osmolarity showed the best performance; errors for all combined-scale calibrated models were below 10%, highlighting the potential of Raman spectroscopy for real-time monitoring of key nutrients, metabolic byproducts, and cell growth parameters in mammalian cell cultures. Wu et al. [41] successfully applied capacitance spectroscopy models to online monitoring by combining variable selection with global calibration methods, achieving real-time prediction of early cell death in mammalian cell cultures. Using an external parameter orthogonalization method, they removed interference from process operations (e.g., feeding), yielding a final model with a root mean square prediction error of 6.56% and thus demonstrating high predictive accuracy. The optimization of culture medium formulations, feeding strategies, bioreactor operations, and scale-up experiments is costly, labor-intensive, and time-consuming. Mathematical modeling frameworks offer the potential to intensify processes while reducing experimental demands.

### 3.3. Technological Innovation

Ongoing improvements in bioreactor technology include the adoption of more efficient gas exchange systems, as well as the optimization of stirring speeds and perfusion systems, to enhance the efficiency and stability of suspension cell cultures. Liang et al. [39] used an online monitoring system to continuously track the chemical parameters of the culture medium, dynamically adjusting tangential flow filtration and alternating tangential flow systems according to cellular metabolic demands. This optimization of perfusion rates enabled efficient production of monoclonal antibodies. Kwon et al. [42] designed and manufactured a high-efficiency microfluidic cell separation device capable of removing small (<10 μm) dead cells and debris from mammalian suspension cultures, thus improving culture quality and increasing biopharmaceutical production efficiency. Given the growing demand for animal cell-based production to support an increasing number of clinical applications, further advancements in bioreactor technology are anticipated in the coming years. The primary objectives are twofold: (1) to increase yields by cultivating animal cells at high densities for the production of high-value protein therapeutics or viral vectors used in clinical gene therapy; (2) to create three-dimensional environments that more closely mimic in vivo conditions needed for tissue or organ regeneration and thus enabling the growth of cell types that are difficult to culture in conventional systems.

## 4. Novel Suspension Cell Lines

The adaptation of adhesion-dependent cells to suspension culture addresses the scale-up limitations of monolayer culture and has become an attractive approach for industrial applications. Multiple cell lines, including MDCK, CHO, Vero, and BHK-21, have been successfully adapted and are now widely used in biopharmaceutical production. Although conventional cell lines such as bovine kidney cells (i.e., MDBK cells) and chicken liver carcinoma cells (i.e., Leghorn male hepatoma [LMH] cells) have also been adapted to suspension culture, they have not yet been adopted for large-scale industrial use. These conventional lines remain in the research stage, and their industrial suitability—including production efficiency and product compatibility—requires further validation. Novel suspension cell lines such as EB66, PER.C6, and AGE1.CR have been developed as promising new substrates for biopharmaceutical production.

### 4.1. EB66 Cells

The EB66 cell line was derived and established from duck embryonic stem cells by Valneva, a French company, and is a passagable suspension cell line. It is the only duck cell line with complete documentation in the U.S. FDA’s Master File for Biological Products and is well-known to regulatory authorities in Europe, Latin America, Asia, and Africa. Several commercial vaccines based on the EB66 platform have been approved for human and veterinary use, including influenza A virus, parvovirus, and avian influenza vaccines [43]. Compared to traditional heterologous cell lines such as MDCK and Vero, the EB66 cell line exhibits higher sensitivity and adaptability to avian viruses. During production, the EB66 cell line enables serum-free, fully suspended high-density culture, offering advantages such as short doubling time, strong viral sensitivity, high safety, low production costs, and no tumorigenicity. This truly achieves large-scale cultivation, making it highly suitable for the scaled-up production of vaccines. Brown et al. [44] reported that EB66 suspension cultures reached a maximum cell density of approximately 3 × 10^7^ cells/mL at 37 °C; they documented good viral titers for influenza viruses and modified vaccinia Ankara (MVA) virus. Léon et al. [45] showed that EB66 cells exhibit high tolerance to MVA virus, supporting their suitability for MVA vaccine production. Nikolay et al. [46] demonstrated that EB66 cells grown in chemically defined medium reached a density of 1.6 × 10^8^ cells/mL. Infection studies with EB66-adapted viruses yielded titers of 7.3 × 10^8^ plaque-forming units (PFU)/mL for yellow fever virus and 1 × 10^10^ PFU/mL for Zika virus, equivalent to approximately 10 million vaccine doses from a single bioreactor run. These findings indicate that EB66 perfusion cultures are effective for vaccine production, particularly for the low cell-specific yields of yellow fever virus. Endo et al. [47] used EB66 cells to produce an H5N1 influenza A vaccine; the addition of AS03 adjuvant further enhanced immunogenicity. The resulting vaccine has shown no adverse reactions to date and could alleviate reliance on chicken embryo-based production, enabling rapid, manufacturing of sufficient influenza A vaccines. Overall, these results demonstrate the feasibility of using the EB66 cell line for vaccine production.

### 4.2. CCX.E10 Cells

CCX.E10 cells are a continuous avian cell line derived from quail embryos and cultured in serum-free suspension medium. Kraus et al. [48] conducted a comprehensive characterization of CCX.E10 cells, confirming that they meet all major regulatory requirements, including growth in chemically defined medium, absence of tumorigenicity, and high viral susceptibility. Göbel et al. [49] described a fully scalable production process for the vesicular stomatitis virus (VSV)–Newcastle disease virus (NDV)–oncolytic virus in CCX.E10 cells. Across multiple production systems and container scales (15 mL to 10 L), high VSV–NDV yields were achieved in batch mode; maximum titers reached 4.2 × 10^8^ 50% tissue culture infectious dose (TCID_50_)/mL. These results indicate that CCX.E10 is a promising host for industrial oncolytic virus production, offering high cell concentrations and scalable manufacturing options in chemically defined media under batch conditions. The ability to consistently achieve high VSV–NDV yields also makes it a potential platform for large-scale production of this and other similar fusion viruses being developed as clinical oncolytic viral drug candidates.

### 4.3. PER.C6 Cells

The PER.C6 cell line is derived from primary human retinal blastoma cells; it was immortalized through transfection with a plasmid expressing adenovirus type 5 E1A and E1B proteins [50]. PER.C6 cells can grow in suspension in serum-free medium and have been confirmed to be free of exogenous agents and reverse transcriptase activity. This cell line is suitable for the proliferation of numerous viruses, including influenza virus and West Nile virus; it can grow at high density in serum-free suspension cultures, making it an attractive option for vaccine production at relatively low cost. Sanders et al. [51] used PER.C6 cells for poliovirus vaccine production, revealing sensitivity to serotypes 1, 2, and 3. Compared with Vero cells, PER.C6 cultures produced higher titers and D-antigen yields, and scale-up from flasks to laboratory-scale bioreactors was easy. Subsequently, Leroux-Roels et al. [52] used PER.C6 cells to produce Sabin strain inactivated poliovirus vaccine (sIPV) without adverse reactions. The PER.C6-derived sIPV demonstrated good tolerability and high immunogenicity in adults with pre-existing poliovirus antibodies. Additionally, PER.C6 cells are suitable for the production and propagation of influenza viruses. For example, Pau et al. [53] showed that PER.C6 cultures in serum-free suspension medium could reach densities of 1 × 10^7^ cells/mL and were susceptible to both influenza A and B strains, producing high-titer viruses within a short time and yielding abundant influenza antigens. In summary, the PER.C6 cell line has certain advantages in terms of production efficiency and cost-effectiveness, particularly in protein production and viral replication. However, its widespread application is limited due to patent protection and licensing fees. In terms of regulatory acceptance, although the PER.C6 cell line is well known to many regulatory agencies, no biological products produced using this cell line have yet been approved for marketing [54]. In contrast, traditional cell lines (such as CHO) have higher recognition and broader application in terms of regulatory acceptance and application scope.

### 4.4. AGE1.CR Cells

AGE1.CR is a continuous cell line derived from primary Muscovy duck embryo cells. The CR cell line was further modified by introducing an expression cassette for the structural gene *pIX* from human adenovirus to enhance viral replication [55]. This modification enabled both AGE1.CR and AGE1.CR.pIX cell lines to proliferate in suspension in culture medium free of animal-derived components and it has low dependence on glutamine during production, which helps reduce the cost of culture media. Lohr et al. [56] investigated the potential of AGE1.CR and AGE1.CR.pIX cells for infection with influenza A viruses (H1N1, H3N2) and for the production of the MVA vaccine through cellular metabolism. Both viruses reached high titers—2100 hemagglutinin units/mL for influenza virus and 1 × 10^8^ PFU/mL for MVA—demonstrating the suitability of this cell line for pharmaceutical-scale influenza and MVA production. Subsequently, the same team showed that AGE1.CR.pIX cells support attenuated live influenza vaccine production, with the strain achieving peak titer 24 h post-infection in chemically defined medium. The AGE1.CR cell line has certain advantages in terms of production efficiency and cost-effectiveness, particularly in virus production. However, its widespread application is somewhat limited due to its relatively low regulatory acceptance. This novel suspension cell line can be used for efficient virus production and has the potential to transform vaccine manufacturing methods. With regard to influenza vaccines, direct virus isolation and seed preparation in continuous cell lines can shorten adaptation time and produce vaccine virus seeds with characteristics more closely resembling the original virus isolates. Further cell modifications—such as introduction of the adenovirus *pIX* gene—may require concurrent viral adaptation to the new host, thus improving the stability and efficiency of vaccine production.

## 5. Applications

### 5.1. Production of Viral Vaccines

**Figure 4:**
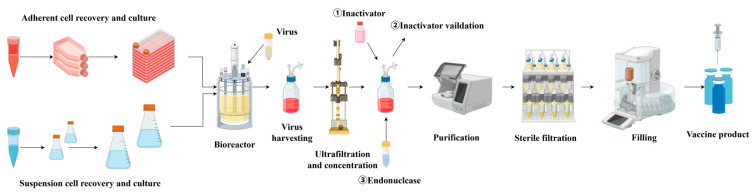
Cell-based viral vaccine production process.

The production of viral vaccines relies on efficient cell culture systems. Suspension cells, which can be cultivated at large scale in bioreactors and can achieve high cell density and viral yields, have become an important substrate for viral vaccine manufacturing (Figure 4). Examples include suspension cell lines such as MDCK, BHK-21, HEK293, *Spodoptera frugiperda* (SF9), and *Trichoplusia ni* (High-Five), which are widely used in the production of vaccines for influenza virus, rabies virus, foot-and-mouth disease virus, and recombinant viral vectors (Table 3).

Silva et al. [81] cultured HEK293-SF suspension cells using a fed-batch continuous harvest or perfusion method, achieving infection rates as high as 9 × 10^6^ cells/mL. The influenza A virus hemagglutinin content obtained was comparable to that reported for other suspension cell lines [82] but lower than that achieved with suspended MDCK cells [57]. Park et al. [61] successfully utilized BHK-21 suspension cells for foot-and-mouth disease virus vaccine production in large bioreactors. To enhance vaccine productivity, a customized medium was developed; during scale-up from 50 L to 200 L bioreactors, comparable cell growth and viral titers were maintained.

Additionally, suspension culture technology can be used for recombinant virus production. For example, Grieger et al. [74] developed a suspension culture-based method for producing recombinant adeno-associated virus (AAV) vectors using HEK293 cell lines, achieving high-titer and high-purity recombinant AAV through optimized transfection conditions and purification strategies. Wang et al. [83] demonstrated that SF9 cells can express the AAV *rep* and *cap* genes using a baculovirus expression vector platform, without requiring auxiliary viral genes beyond those provided by the expression vector. This approach reduced exogenous DNA contamination and improved the ratio of intact particles to empty particles. In summary, suspension culture technology has been widely applied and undergone rapid advancement in the production of vaccines, particularly inactivated viral vaccines. With continued progress in biotechnology, the application of suspension culture technology in inactivated vaccine manufacturing is expected to expand further. Future developments may include optimization of culture medium formulations, increases in cell density, and enhancements in viral yield, combined with advanced bioreactor technologies to enable more efficient vaccine production. However, in large-scale manufacturing, the maintenance of consistent product quality and stability without excessive production costs will remain important challenges to address.

### 5.2. Recombinant Protein Expression

Antibody-based therapeutics and recombinant protein reagents are typically produced in mammalian expression systems, which provide human-like post-translational modifications. Among mammalian cell lines used for recombinant protein expression, CHO cells are the most common due to their ease of cultivation, suitability for high-yield clone selection and gene amplification, and ability to produce high protein yields. They are genetically stable, readily transfected, and facilitate both transient and stable expression. Liu et al. [80] established an efficient transient transfection system to evaluate the production of therapeutic proteins, including human erythropoietin and human coagulation factor IX, in HEK293 and CHO cells. Both cell types demonstrated high transfection efficiency and high protein yields in serum-free suspension culture. However, because CHO cells generate non-human post-translational modifications, alternative or complementary cell lines are required to address this limitation. Human cell lines such as HEK293 can produce therapeutic proteins with native post-translational modifications. Dash [81] proposed a method for rapidly generating stably transfected HEK293 suspension cell lines for recombinant protein production; this method showed substantial yield improvements and functional validation in the production of the severe acute respiratory syndrome coronavirus 2 receptor-binding domain. Chin et al. [27] developed a stable recombinant protein production system in HEK293 cells by knocking out the *GLUL* gene using CRISPR/Cas9 and selecting high-expression cell pools with methionine sulfoximine. This approach enabled high-titer production of recombinant human erythropoietin without heterologous protein contamination, thus providing an efficient, stable, and clinically compliant platform for the production of complex recombinant proteins. Such systems are expected to become preferred platforms for future recombinant protein manufacturing.

Suspension cell systems offer substantial advantages for recombinant protein production, including the ability to maintain stable protein expression through optimized selection and culture conditions. Additionally, HEK293 cells can perform humanized glycosylation, reducing immunogenicity. Nonetheless, challenges remain, such as improving gene integration efficiency, adapting cells to serum-free conditions, and reducing the high costs of large-scale production. Future progress in the development of novel selection markers, as well as advancements in media formulations and culture optimization, is expected to enable broader and more cost-effective use of suspension cells in recombinant protein production.

### 5.3. Monoclonal Antibody Production

Since Georges Köhler and César Milstein developed hybridoma technology and were awarded the Nobel Prize in 1975, the demand for monoclonal antibodies for therapeutic and diagnostic applications has continued to grow. Suspension cell culture is an efficient and scalable method for monoclonal antibody production, particularly suitable for large-scale biopharmaceutical manufacturing. Masuda et al. [82] established a new CHO cell line, CHO-MK, which exhibited significantly shorter doubling times than conventional CHO cell lines after adaptation to serum-free suspension culture conditions. This cell line produced a model monoclonal antibody, immunoglobulin G1 (IgG1), reaching a concentration of approximately 5 g/L by day 8 in a fed-batch culture using a 200-L bioreactor. Komolpis et al. [83] achieved high-volume production of a monoclonal antibody specific for enrofloxacin using a perfusion culture mode in a stirred-tank bioreactor equipped with a rotating filter, yielding 61.4 mg/day with cell viability reaching 1.57 × 10^6^ cells/mL in 5 days.

Suspension cell-based monoclonal antibody production offers advantages such as high efficiency, scalability, cost-effectiveness, and high product quality. It also reduces contamination risks, making it ideal for industrial-scale manufacturing. With continued technological advancements and process optimizations, both the efficiency and quality of monoclonal antibody production in suspension cell systems are expected to improve further.

## 6. Conclusions

Serum-free, fully suspended cell culture has become a key technology in biomanufacturing due to its substantial advantages. By eliminating serum components, it effectively reduces batch-to-batch variability, pathogen contamination risk, and immunogenicity, thus improving culture stability and product quality. Additionally, serum-free media with well-defined chemical compositions, as well as the scalability of suspension culture, considerably lower production costs; this approach is widely adopted for industrial production of recombinant proteins, monoclonal antibodies, and inactivated viral vaccines. However, multiple challenges remain. Adaptation of certain cell lines to serum-free suspension conditions can be difficult; some exhibit slow growth, high apoptosis rates, and unstable product expression. Furthermore, the development of serum-free media requires cell type-specific optimization, leading to long research and development cycles and high costs. Additional limitations include fluctuating nutrient requirements during high-density culture, cytotoxicity from accumulated metabolic waste, and inefficiencies in mass and heat transfer during scale-up, all of which restrict further gains in productivity and quality.

Looking ahead, advances in synthetic biology will enable targeted modification of cellular metabolic pathways to enhance adaptability to serum-free environments and increase expression levels of desired products. Continuous production systems integrated with real-time online monitoring technologies will allow dynamic process control, thereby reducing variability and improving efficiency. The development of novel bioreactor designs, such as microfluidic chips and intelligent temperature-controlled reactors, will further optimize mass and heat transfer, overcoming bottlenecks in high-density culture. Serum-free, fully suspended cell culture technology is expected to drive transformative advancements in the biopharmaceutical and cell therapy industries, providing reliable and efficient technical support for personalized medicine and novel drug development.

## Figures and Tables

**Table 1:** Development and characteristics of serum-free media [17].

Type	Characteristics
SFM	Contains various biological materials that can replace serum function, including numerous plant- and animal-derived proteins and undefined components (e.g., bovine serum albumin or α- or β-globulin), used as supplements.
XFM	Contains human-derived components, such as human serum albumin, but excludes animal-derived supplements.
AFM	Free of animal-derived proteins; required proteins are obtained from recombinant sources or proteolytic digestion.
PFM	Serum-free, with no protein or low protein content; contains undefined components such as peptides (hydrolyzed proteins).
CDM	Serum-free, protein-free; contains only chemically defined components; suitable for diverse cell types and recombinant protein production.

Abbreviations: AFM = animal-free media, CDM = chemically defined media, PFM = protein-free media, SFM = serum-free media, XFM = xeno-free media.

**Table 2:** Genetically engineered cell lines developed to enhance suspension growth capacity.

Cell Line	Gene	Operation	Function	Reference
CHO	*Igfbp4*	Downregulation	Shortened the time required for CHO-K1 cells to transition from adherent to suspension culture.	[22]
	*FUT8*	Knockout	Knockout cell lines exhibited greater anti-apoptotic ability compared with wild-type CHO-S cells.	[23]
MDCK	*Siat7e*	Insertion	Stable expression of *Siat7e* enabled adaptation of MDCK cells to suspension culture.	[24]
	*CLDN1*	Downregulation	Overexpression of miR-175 and knockdown of *CLDN1* facilitated establishment of suspension-adapted MDCK cells.	[25]
HEK293	*NGFR*	Knockout	*NGFR* knockout enabled establishment of suspension-adapted HEK293 cells.	[26]
	*GLUL*	Knockout	*GLUL* knockout further improved the suspension growth characteristics of cells.	[27]
Vero	*CDH18*	Downregulation	Key gene potentially involved in Vero cell adaptation to suspension culture.	[28]
	*PTEN*	Upregulation	Knockdown of *PTEN* significantly reduced adhesion, facilitating establishment of a suspension cell line.	[29]

**Table 3:** Suspension cell lines available for viral proliferation.

Suspension Cell Line	Virus	References
MDCK	Influenza A virus	[57]
BHK-21	Duck Tembusu virus, Japanese encephalitis virus, foot-and-mouth disease virus, rabies virus	[58,59,60,61]
MDBK	Bovine viral diarrhea virus, foot-and-mouth disease virus	[62,63]
CHO	Severe acute respiratory syndrome coronavirus 2 (SARS-CoV-2)	[64]
Vero	Influenza viruses, Zika virus, rabies virus, SARS-CoV-2	[18,65,66,67]
PK-15	Pseudorabies virus, classical swine fever virus	[68,69]
AGE1.CR.pIX	Fusogenic oncolytic virus, influenza A virus, MVA virus	[70,71,72]
EB66	Duck Tembusu virus, Zika virus	[46,73]
CCX.E10	Fusogenic oncolytic virus	[49]
PER.C6	Poliovirus, influenza A virus (high titer)	[52,55]
HEK293	Recombinant adenovirus, rabies virus, SARS-CoV-2	[74,75,76]
SF9	Recombinant adenovirus, recombinant baculovirus	[77,78]
High-Five	Influenza A virus, recombinant baculovirus	[79,80]

## Data Availability

The data supporting the findings of this review (e.g., the extracted dataset and the source data for newly created figures and tables) are available in the [Mendeley Data] repository at [Reserved DOI: https://doi.org/10.17632/p2tg48xy2k.1].
